# Engineered Nanomaterials in Food: Implications for Food Safety and Consumer Health

**DOI:** 10.3390/ijerph110605720

**Published:** 2014-05-28

**Authors:** Alina Martirosyan, Yves-Jacques Schneider

**Affiliations:** 1 Laboratory of Cellular, Nutritional and Toxicological Biochemistry, Institute of Life Sciences (ISV) & UCLouvain, Louvain-la-Neuve B1348, Belgium; 2 Interdisciplinary Nanoscience Center (iNano), Aarhus University, 8000 Aarhus C., Denmark; E-Mail: alina_mart@list.ru

**Keywords:** nanotechnology, engineered nanomaterials, nanofood, metal-based nanoparticles, exposure, toxicity, consumer safety

## Abstract

From the current state-of-the-art, it is clear that nanotechnology applications are expected to bring a range of benefits to the food sector aiming at providing better quality and conservation. In the meantime, a growing number of studies indicate that the exposure to certain engineered nanomaterials (ENMs) has a potential to lead to health complications and that there is a need for further investigations in order to unravel the biological outcomes of nanofood consumption. In the current review, we summarize the existing data on the (potential) use of ENMs in the food industry, information on the toxicity profiles of the commonly applied ENMs, such as metal (oxide) nanoparticles (NPs), address the potential food safety implications and health hazards connected with the consumption of nanofood. A number of health complications connected with the human exposure to ENMs are discussed, demonstrating that there is a real basis for the arisen concern not only connected with the gut health, but also with the potency to lead to systemic toxicity. The toxicological nature of hazard, exposure levels and risk to consumers from nanotechnology-derived food are on the earliest stage of investigation and this review also highlights the major gaps that need further research and regulation.

## 1. Introduction

Engineered nanomaterials (ENMs) designed for use in many commercial materials, devices and structures are already found in various common products—sunscreens, cosmetics, sporting goods, clothing, tires, electronics, *etc*. Nanotechnology applications also extend to techniques like drug delivery, diagnosis, biomedical imaging, ground water remediation, and so forth [1]. 

The massive industrial production and application of ENMs currently and the predicted increase in the near future may result in their appearance in various environments, yielding the possibility of human exposure to these ENMs through inhalation, dermal contact, or ingestion. The annual release of ENMs into the environment cannot be accurately estimated due to the rapidly increasing production volumes, lack of knowledge on the amount of ENMs applied in and released from different applications and products. Due to high-volume production of consumer products containing ENMs, such as nanoparticles (NPs) of silver, titanium dioxide, zinc oxide, silica, *etc.* human exposure to these man-made NPs is possible directly (via personal healthcare products, cosmetics, food, water, drinking, drugs and drug delivery system) and/or indirectly, e.g., through the release of these compounds into the environment [2,3,4]. The latter may potentially result in the contamination of drinking water and uptake into the human food chain [5]. 

The dietary consumption of NPs in developed countries is estimated more than 10^12^ particles/day, consisting mainly of TiO_2_ and mixed silicates [6]. In some countries, ENMs are already used in food supplements and food packaging, with nanoclays as diffusion barriers and Ag NPs as antimicrobial agents [7,8]. ENMs likely found in nanofood products fall into three main categories: inorganic, surface functionalized materials, and organic ENMs [7]. Inorganic nanomaterials, which will be mainly discussed in this review, find currently their applications in food industry (food additives, food packaging or storage) and include ENMs of transition metals (e.g., silver, iron, titanium, and zinc), alkaline earth metals (e.g., calcium and magnesium); and non-metals (e.g., selenium and silicates).

While the successful implementation of nanotechnology is important for the growth of the global economy, there is also a need to consider the possible environmental health and safety impact of these ENMs that could lead to hazardous biological outcomes. Once in the environment, ENMs may undergo diverse physical, chemical, and biological transformations (e.g., deposition, adsorption, agglomeration, aggregation, oxidation/reduction reactions, (bio)functionalisation), potentially altering their biological impact and fate [5,9]. Certain local environmental factors, such as pH, salinity, microbes, natural organic matter, *etc.* may affect the reactivity, mobility, and toxicity of ENMs [10]. 

An area that could highly benefit from nanotechnology is the food industry with big potentials for food safety, quality, and preservation (shelf life extension) [11,12]. In the food sector, the uses of nanotechnology-derived food ingredients, additives, supplements and contact materials are expected to grow rapidly. Nanotechnology analysts estimated that between 150–600 nanofoods and 400–500 nanofood packaging applications are already on the market [13]. According to the Project on Emerging Nanotechnologies, as of March 2013, in the category “Food and Beverage” are indicated 204 products, while according to potential exposure pathways into the human body from a theoretical perspective there are 107 products with a potency to be ingested [14]. Chaudhry *et al*. [7] claim that more than 200 companies worldwide are conducting research and development on the use of nanotechnology in agriculture, engineering, processing, packaging, as well as for delivery of food and nutritional supplements.

Application of ENMs in agri-food industry may pose new indirect sources of food contamination, as may arise from e.g., nano-sized pesticides and veterinary medicines, contact of food with nanoparticulate-based coatings during preparation or processing, or potential migration of ENMs from food packaging. There are already known examples of pesticide formulations that are based on microemulsion or microencapsulation technology [7]. A literature review on nenopesticides was published recently that combines the existing information and concludes that the nanoformulations expected to have significant impacts on the fate of active ingredients and/or introduce new ingredients for which the environmental fate is still poorly understood (e.g., Ag·NPs) [15]. Considering the lack of the knowledge of the environmental behaviour and the fate of ENMs, it is difficult to assess whether ENMs may bioaccumulate in the food chain.

Numerous food-related applications of ENMs that have the potential to be directly or indirectly (e.g., after mucociliary clearance from the respiratory tract after being inhaled [16]) ingested make an issue of current concern the study of the potential adverse health effects of ENMs on the gastrointestinal tract (GIT). The whole cascade of events including absorption, distribution, metabolism and excretion/elimination (ADME) that occur following ingestion determines the internal exposure and toxicity of ENMs. The picture becomes more complicated due to the interactions of nanomaterials with surrounding matrix (GI fluids, food matrix, microflora) and unexpected effects resulting from this. From this point of view, the interaction of NPs with food components is another aspect that may need consideration and about which little information is currently available. The possible interaction of food components may alter the physicochemical properties of ENMs that in turn may influence their passage through the GIT, their ADME properties.

ENMs, due to their specific physicochemical properties and high reactivity, can influence basic cellular processes, such as proliferation, metabolism, and death. Individual ENMs may lead to one or more toxicity endpoints, resulting in dysfunction of these basic processes. In recent years, several *in vitro* studies have assessed the potential adverse health effects of ENMs, pointing out their ability to induce oxidative stress, release toxic ions, disrupt electron/ion cell membrane transport activity and cause oxidative damage and lipid peroxidation, while results from *in vivo* studies have shown that these materials can induce adverse effects on the respiratory, cardiovascular and nervous systems [17,18]. The most relevant pathogenetic pathway linking ENM exposure to tissue damage is represented by the induction of reactive oxygen species (ROS) generation [19]. The latter may modulate intracellular calcium concentrations, activate transcription factors, and induce cytokine production [20]. Other common toxicity endpoints involve cytotoxicity, genotoxicity, stimulation of an inflammatory and/or immune response [21]. 

In this review, we will provide information on the probable sources of food contamination with ENMs, available toxicity profiles for the ENMs commonly applied in the food industry (metal (oxide) NPs), the existing data on the possible health complications on ingested NPs and the data gaps existing currently in this area. 

## 2. Applications of ENMs in the Food Sector

Food itself contains many nanostructured materials. This is important to note since the distinction between natural nanostructures and deliberately ENMs produced to a particular specification is not always clear. Food and feed ingredients comprise many components, including biopolymers such as proteins, complex carbohydrates and fats, with sizes extending down to the nanoscale (e.g., casein, alginic acids and micelles/foams/colloids). Natural nanostructures are not regarded as products of nanotechnology, and they need to be differentiated from deliberately ENMs when considering regulatory requirements and definitions [22].

A number of recent reports and reviews have identified the current and short-term projected applications of nanotechnologies in the food sector [23,24,25,26,27,28]. 

There are already identified potential uses of nanotechnology in virtually every segment of the food industry (Table 1) with four key focus areas:

(i) agriculture-pesticide, fertilizer or vaccine delivery; animal and plant pathogen detection; and targeted genetic engineering,
(ii) food processing-encapsulation of flavor or odor enhancers; food textural or quality improvement; new gelation or viscosifying agents,
(iii) food packaging-pathogen, gas or abuse sensors; anticounterfeiting devices, UV-protection, and stronger, more impermeable polymer films,
(iv) nutrient supplements-nutraceuticals with higher stability and bioavailability.

**Table 1:** (Potential) applications of nanotechnology in food science.

Area of application	Application	Reference
**Agriculture** (Nano-modification of seed and fertilisers/pesticides)	Pesticides	[27]
	Targeted genetic engineering	[29]
	Preservation	[29,30]
	Agrichemical delivery	[29]
	Sensors to monitor soil conditions	[31]
**Processing** (Interactive smart food)	Nanoencapsulation of flavors/aromas	[32,33,34,35,36,37]
	Nanoemulsions	[38]
	Anti-caking agents	[39]
**Nutrition** (Food fortification and modification)	Nutraceuticals	[40,41,42]
	Nutrient delivery	[32,41]
	Mineral and vitamin fortification	[43,44]
	Drinking water purification	[45]
	Sensory characteristics of supplements	[46,47,48,49,50,51]
**Products** (Smart packaging and food tracking)	UV protection	[52,53,54]
	Antimicrobials	[55,56,57,58,59,60,61,62,63]
	Condition and abuse monitors	[46,47,64]
	High barrier plastics	[65,66,67,68]
	Security	[45,69,70]
	Contaminant sensors	[51,71,72,73,74,75]

As it can be seen from Table 1, the potential for food nanotechnology applications seems unlimited. All facets of the food industry from ingredients to packaging and food analysis methods are already looking into nanotech applications. These are resulting in numerous promising applications for improved food production, processing, packaging, and storage [32,76,77], as well as targeted nutrient delivery systems [78]. Bacteria identification and food quality monitoring using biosensors; intelligent, active, and smart food packaging systems; nanoencapsulation of bioactive food compounds are a few examples of emerging applications of nanotechnology for the food industry. Perhaps the most advanced investigations into the use of ENMs in food applications are in food contact materials for the benefit of maintaining (e.g., the use of silica nano sheets to enhance barrier properties, the inclusion of anti-microbials such as nanosilver) or monitoring (using microbial sensors) the quality and safety of foods [22].

Another major focus of current nanotechnology application in food is the processing and formulation of food ingredients to form nanostructures that are claimed to offer improved taste, texture and consistency [79], enhanced bioavailability [80] and allow mixing of “incompatible” ingredients in food matrix [81]. Different types of functional nanostructures can be used as building blocks to create novel structures and introduce new functionalities into foods, such as nanoliposomes, nanoemulsions, NPs and nanofibers [79,82].

An example of the soluble nanomaterial under development is nanosalt, enabling consumers to lower their salt intake, since a small amount will cover a larger area of the food surface [83]. Nanotechnology would even be used to manufacture “smart” packaging to dramatically extend the shelf life of food and enable it to be transported even further [14]. Smart packaging (containing nanosensors and antimicrobial activators) is being developed that will be capable of detecting food spoilage and releasing nanoantimicrobials to extend food shelf life, enabling supermarkets to keep food for even greater periods before its sale. Nanosensors embedded into food products as tiny chips invisible to the human eye would also act as electronic barcodes [13].

## 3. Fate of ENMs Following Ingestion

Whether it exists as nanostructured food ingredient, nanocarrier or nano-sized particles incorporated in food packaging, human exposure to ENMs present in food or food contact materials occurs through ingestion. Owing to the huge surface area of the GIT, ingestion is probably the most common way of intentional exposure to various NPs. There is currently not much information regarding metabolism/biotransformation of ENMs upon oral administration in human model. In this relation, there are several concerns, such as those connected with the small sizes of ENMs that may enter the food chain undetected, accumulate within tissues and organs, and taken up by individual cells [81]. There is also concern that the introduction into food of ENMs designed to carry dietary supplements could also lead to introduction of foreign substances into the bloodstream [7]. One of the different complications as a result of nanofood ingestion is the possibility of altered absorption profile and metabolism in the body. The latter could occur as a result of several processes:

1. As ENMs have been shown to have a greater ability to cross the gut wall, as compared to microparticles of the same kind, their enhanced absorption and bioavailability would result in higher internal exposure (higher plasma concentrations). The nanofood may also alter the way by which food ingredients are distributed or behave in the GIT [7].
2. It is not only the bulk material of the ENM that may trigger biological effects. ENMs may form a biofunctional corona and thus act as carriers of these substances to different biological tissues, potentially influencing the absorption of molecules, e.g., by introducing unintended molecules across the GIT and lead to unpredictable effects, known as “Trojan horse” effect [83].
3. In addition, it has been shown that the consumption of food containing NPs has the potential to alter body metabolism of experimental animals. For instance, oral administration of nanocalcium-enriched milk has been shown to alter the calcium metabolism in rats [84].

**Figure 1:**
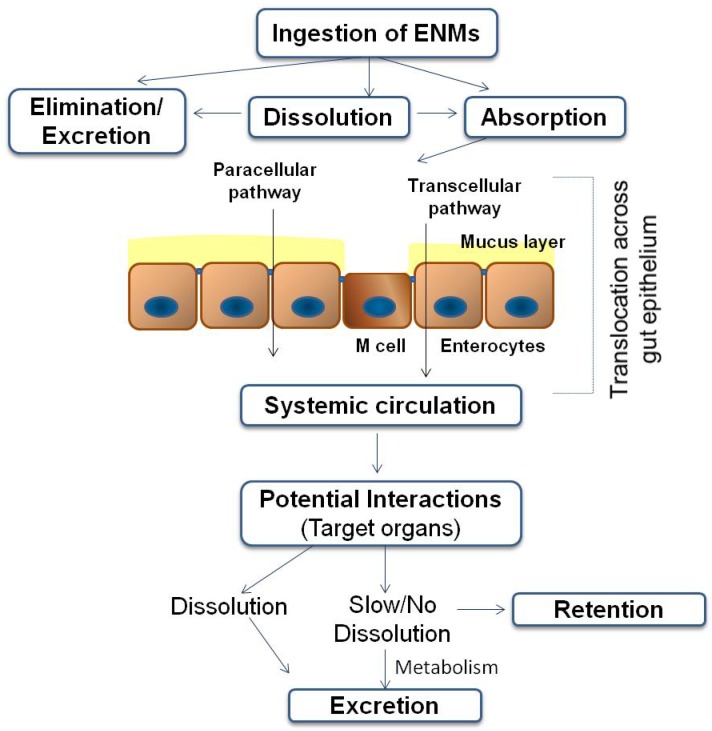
The fate of food-related ENMs in the GIT (modified from Cockburn *et al*. [22]).

ENMs entering the body through ingestion are subject to digestive processes in the GIT. The GIT and its mucosal layer should play the role of a selective barrier to systemic exposure of materials, including particulate matter, in which case the ENM may remain in the gut lumen, perhaps with a potential for interaction with GIT surfaces or with inhabitants of the lumen (e.g., microbiota), but essentially being fully eliminated from the body via the faeces. The behavior of ENMs entering the body through ingestion are described in [85] and schematically presented in Figure 1.

The GIT is also covered by a protective layer of mucus, *i*.*e*., a complex network of highly branched glycoproteins and macromolecules. The importance of this mucosal layer as a barrier to ENMs uptake (and so also for consideration in any *in vitro* testing methodology) has been demonstrated [86,87]. Absorption of particulate material occurs across the GIT (mainly in the small intestine) primarily via transcytosis in the M-cells of the Peyer’s Patches in the gut-associated lymphoid tissue, and has been described for the uptake of NPs (20–100 nm) and small microparticles (100–500 nm) [88].

Translocation through the gut epithelium leads to internal systemic exposure, where ENMs could be distributed throughout the body, gain access to other internal body compartments with further internalization and retention in cells and organelles. For example, an ENM may enter the bloodstream via portal circulation to the liver or via mesenteric lymph nodes into the lympho-reticular system, enabling transport throughout the body and to other organs.

It is accepted that the physicochemical characteristics of ENMs are the key determinants of their techno-biological functionality, as well as the source of potential adverse health effects. A list of such physicochemical properties has been defined by the Organisation for Economic Co-operation and Development (OECD) [89]. Amongst these parameters the size, chemical composition aggregation/agglomeration state, and surface treatment/coating of ENM appear to be most critical for nanotoxicity issues [90]. Solubility is another key physicochemical property that warrants particular consideration, where insoluble or only partially soluble in the digestive fluids ENMs are paid more attention due to their potential to cross the GIT as an intact particle. As stated by the EU Scientific Committee on Emerging and Newly Identified Health Risks (SCENIHR), free and low solubility ENMs are a priority concern for human and environmental safety [91]. 

Another important issue to be considered in the assessment of hazardous effects of ENMs to human health and the environment is the understanding of how the ENM was manufactured. Nanoscale impurities may arise from the manufacturing process, where changes such as the use of different solvents, time/temperature conditions and changes to the starting chemicals (e.g., alternative starting materials, different purity levels or concentrations of chemicals) may influence the types and/or quantities of impurities in the final product [92]. Surfactants can drastically change the physicochemical properties of ENMs, such as magnetic, electric, optical properties and chemical reactivity [93,94,95,96], thus affecting their cytotoxicity. It was shown that NPs coating with various types and concentrations of surfactants before injection significantly affects their distribution in the body [96]. Surface coatings can render harmful particles non-toxic while less harmful ones can be made highly toxic. Hence, additional agents, e.g., dispersing agents and surface modifiers often used in the manufacture of ENMs should be considered in the safety assessment of ENMs.

In the GIT the physicochemical properties of ENMs may change as a result of their interaction with food, digestive enzymes, electrolytes, intestinal microbiota, *etc*. [97,98,99]. In addition, pH varies in different segments of the GIT, which may change the reactivity and toxicity of the particles. When in biological fluids, whether these are fluids of the GIT or a culture microenvironment, ENMs will develop a corona of adsorbed proteins, small molecules, and ions [100,101,102]. The physicochemical properties of such an ENM-protein complex often differ significantly from those of the pristine ENM, which in turn reflects on the subsequent biological responses that are evoked in cells and tissues, and on biodistribution profiles of the ENMs in the body [101,103]. For instance, corona formation may lead to decreased cytotoxicity, possibly by decreasing the cellular uptake of NP [104] or mitigating cell membrane damage [105]. Therefore, it is generally accepted that the protein corona, rather than the pristine NP itself, defines the biological identity of NPs [101,106,107].

It worth noting that the body also produces nanomaterials *de novo* in the gut lumen. For example calcium phosphate NPs (20–200 nm) are formed in the mid-distal aspect of the small intestine due to homeostatic secretion and co-precipitation of calcium and phosphate ions [108]. Van der Zande *et al*. [109] have shown the *in vivo* formation of Ag NPs from silver ions probably composed of silver salts. This possibility should also be taken into account when assessing the toxicity profiles of ENMs, especially in the case of materials that tend to release ions, as these ions may form NPs in the intestine and thus lead to unpredictable health complications. 

## 4. Food-associated ENMs: Metal (Oxide) NPs and Their Toxicity Profiles

ENMs that are considered to be likely ingested may be separated into several main categories–metals and metal oxides, carbon-based materials (e.g., fullerenes, carbon nanotubes), polymeric/dendrimeric materials and liposomes. Among these categories, the metal/metal oxide NPs presently have the highest potential to be ingested due to their increasing inclusion in dietary supplements and food conservation materials [24,110,111]. Currently, metal (oxide) NPs have not been comprehensively assessed in regard to the potential effects on human health. Below we will discuss the existing information on hazard identification of mostly applied metal-based NPs on the GIT based on both *in vitro* cell-based assays and *in vivo* animal experimentation. 

### 4.1. NanoSilver

Ag NPs are currently used in more manufacturer identified products than any other nanomaterial [112]. There are (as of November 2013) at least 390 products that utilize some form of nanosilver for their function [113]: textiles (socks and linens), cosmetics/hygiene products (toothpastes, make-ups), appliances (washing machines and refrigerators), cleaning agents (detergents, soaps), kitchen supplies (food storage containers, bakeware, cutting boards), toys and building materials (paints, caulks, glues). The use of Ag NPs as an antimicrobial, antiodor, and a health supplement has already surpassed all other ENMs currently in use in different sectors [112,113].

Currently very little work has been done to assess the ability of NPs in general, and Ag-NPs in particular, to migrate from the packaging material into the food. Emamifar *et al*. [114] revealed evidence of migration from packaging materials (orange juice package) incorporating Ag or ZnO antimicrobial NPs into a food substance. Ag NPs being incorporated into cellulose pads (fresh beef packaging) are found to lead to detectable levels of silver ions (Ag^+^) leeched into the meat exudates (though not into the meat itself) [115]. There is thus a current need to study the relationships between particle characteristics, polymer type, food pH/polarity and environmental conditions relevant to food production, storage and packaging (e.g., temperature, pressure, humidity, light exposure, storage time). This information will lead to a better understanding of human exposure levels and ease the assessment of important aspects of the safety of Ag NP-based food contact materials.

There is growing evidence that Ag NPs are highly toxic to mammalian cells and human health [116,117]. Ag NPs have been shown to damage brain [118], liver [119] and the GIT [120] cells, have specific effects on mitochondria and induce oxidative stress [119]. Ag NPs have been shown to damage cells derived from human and mammalian skin, liver, lung, brain, vascular, and reproductive tissues when evaluated *in vitro* [121]. At high doses, they have been shown to compromise the blood-brain barrier and cause neurotoxicity in rats and mice [122,123,124], as well as the integrity of the gut barrier [121]. Ag NPs at low concentrations *in vitro* caused changes to the cell cycle progression of human hepatoma cells, whereas at higher concentrations induced abnormal cellular morphology, cell shrinkage, and chromosomal damage to a much worse extent than that caused by similar Ag^+^ concentrations, indicating that the toxicity of Ag NPs is not only caused by ion release [125]. In the recent study aimed at unraveling the toxicity of Ag-NPs to GIT cells, it was also shown that the release of Ag^+^ is not sufficient to explain the toxic effects of Ag-NPs [120]. However, there are very controversial data in this relation and there is a need of more thorough investigation with consideration of environmental conditions, e.g., the presence of complex food matrix, temperature, *etc*. 

While there is a growing number of *in vitro* studies showing that Ag NPs are cytotoxic to a variety of mammalian cell types, *in vivo* studies that have investigated the systemic effects of Ag NPs upon exposure by oral route are more ambiguous. For example, though Ag NPs were found distributed in virtually every organ of rats fed a steady NP diet, there were few toxic effects observed except at the highest concentrations [126,127]. An oral intake study in weaning pigs showed Ag NP accumulation in the liver with no acute toxic effects revealed [128]. In addition, lymphocyte infiltration and inflammation has been observed in the livers of mice fed nano- and micro-sized Ag particles, an effect that was exacerbated when particle diameter was on the nanoscale [129]. Van der Zande *et al*. [107] have revealed that Ag NPs were present in practically all organs with the highest levels in the liver and spleen. Silver concentrations in the organs were highly correlated to the amount of Ag^+^ in the Ag NPs suspension, indicating that mainly Ag^+^, and to a much lesser extent Ag NPs, passed the intestines in the Ag NP exposed rats. The authors also demonstrated a long retention of silver in brain and testis, which should be further considered in a risk assessment of these NPs.

Given the still small number of *in vivo* toxicological studies for Ag NPs, a limited generalized conclusion on the effects of Ag NP exposure via food-relevant routes could be done. It is, for example, still unclear: (i) to what extent the biochemical pathways facilitating the processing of Ag^+^ apply to Ag NPs; (ii) to what extent NPs pass through the intestinal lining intact or as dissolved Ag^+ ^due to the highly acidic environment of the stomach; and (iii) to what extent Ag NPs can pass through natural biological barriers such as the gut epithelium, blood-brain barrier, the placenta or get into the breast milk. There is also a knowledge gap concerning the relationship between NP characteristics (size, shape, charge, coating, *etc*.) and toxicity.

### 4.2. NanoTitanium

TiO_2 _is in the top five of NPs used in consumer products [130], accounting for 70% of the total production volume of pigments [131] and consumed annually at about 4 million tons worldwide [132]. TiO_2_ can be used in paints, coatings, plastics, papers, inks, medicines, sunscreens, pharmaceuticals, cosmetics, toothpaste, nutritional supplements and food products, e.g., colorants [39,133,134,135,136,137,138]. These NPs has been the subject of recent efforts to develop effective carriers that enhance the oral uptake of drugs and vaccines [139]. Smaller TiO_2_ particles have higher UV-blocking properties, which can be advantageous for food storage [140], as well as nanosized TiO_2_ prevents microbial growth [141]. TiO_2_-coated packaging film has been shown to considerably reduce *E*. *coli* contamination of food surfaces [142] and has also been shown to disinfect water from fecal coliform [143]. A recent study found that candies, sweets and chewing gums contained the highest amount of TiO_2_ in the scale of <100 nm [144]. TiO_2 _in anatase (E171) is commonly added to granular and powdered foods as anti- caking agents [145]. 

There is no evidence of Ti being an essential element for human beings or animals. The Ti compound concentration in drinking water is generally low. A typical diet may contribute 300–400 μg/day [146]. TiO_2_ particles are produced and used in varying particle size fractions including fine (approximately 0.1–2.5 μm) and nanosize (<0.1 μm, primary particles) [147]. Human exposure to TiO_2_ NPs may occur during both manufacturing and use. The U.S. Food and Drug Administration (FDA) approved TiO_2_ as a food color additive with the stipulation that the additive should not to exceed 1% w/w. TiO_2_ was also approved by the US FDA in food packaging [148]. In the “Risk Assessment of Manufactured Nanomaterials TiO_2_ Executive Summary” compiled by the New Energy and Industrial Technology Development Organization (NEDO) in Japan, the acceptable exposure concentration of TiO_2_ NPs was estimated to be 1.2 mg/m^3^ for an 8 h workday and a 40 h workweek [149]. 

The various fields of TiO_2_ application makes favorable the oral exposure to these NPs. Wang *et al*. [134] investigated the distribution and acute toxicity of TiO_2_ nano- (25 and 80 nm) and microparticles (155 nm) in mice, following oral exposure (5 g/kg). According to the Scientific Committee on Consumer Safety (SCCS) Opinion from 22 July 2013 on nanotitanium revised on 22 April 2014, the TiO_2_ NPs (anatase/rutile mixtures) showed LD_50_ in rat >2150 mg/kg leading to low acute oral toxicity [150]. TiO_2_ NPs have been shown to be absorbed from the GIT into the blood and distributed to the liver, spleen, lungs and kidneys [134]. These NPs are highly stable and thus not degraded in the intestine. They are, therefore, typically taken up by M-cells of Peyer’s patches and passed to underlying macrophages. As macrophages are also unable to digest the particles, it is common to see pigmentation in cells at the base of human intestinal lymphoid aggregates due to particle accumulation [151].

TiO_2_ NPs have been linked with potentially adverse health effects in some studies. For instance, it was found that mice fed certain kinds of TiO_2_ with their drinking water for 5 days exhibited DNA and chromosomal damage and inflammation [136]. Studies on TiO_2_ NPs have shown that it destabilizes the cell membranes of digestive gland tubes *ex vivo* [152]. Recently, a review discussed very well the *in vitro* and *in vivo* toxicity effects of TiO_2_ [146]. From the available so far data, it is clear that TiO_2_ NPs can be absorbed through the lung or GIT into systemic circulation and then distributed in different organs such as liver, kidneys, spleen, or even brain causing localized effects. However, the rate of such translocation is currently uncertain. TiO_2_ NPs may have the potential to penetrate the blood-brain, blood-testis and blood-placenta barriers. However, the rate of translocation appears low and evidence is lacking that could link systemic responses to translocation of particles to target sites. 

Many studies have been conducted *in vitro* and *in vivo* to investigate the genotoxicity of TiO_2_ NPs, but the results are conflicting and doses employed were high. Certain reproductive and developmental toxicities in experimental animals or cell cultures have been observed in a few *in vivo* and *in vitro* studies. Whether human exposure to TiO_2_ NPs causes reproductive and developmental toxicities is unclear. In addition, TiO_2_ NPs induced oxidative stress and alterations in cell signal transduction pathways that may play an important role in the etiology of TiO_2 _NP-induced carcinogenesis of TiO_2_ at relatively high doses. However, these studies should be repeated at doses relevant to normal occupational or environmental exposure conditions. Therefore, further investigations are needed to elucidate the molecular mechanisms of toxicity of TiO_2_ NPs, as well as their systemic toxicity when exposed to real-world doses both acute and chronic.

### 4.3. NanoZinc

ZnO is another commonly used particle with similar utility to TiO_2_. ZnO NPs are widely used in various applications including cosmetics, paints, as drug carriers and fillings in medical materials [153]. ZnO exhibits antibacterial activity that increases with decreasing particle size [154], which could be stimulated by visible light [155]. ZnO NPs have been incorporated in a number of different polymers including polypropylene [156]. In addition, ZnO effectively absorbs UV light without re-emitting it as heat and therefore improves the stability of polymer composites. ZnO is also used in nutritional supplements such as multivitamins [43,44].

Exposure of RKO and Caco-2 human colon carcinoma cells to ZnO NPs (8–10 nm) revealed changes in metal metabolism, chaperonin proteins, and protein folding genes after gene profiling, without a pro-inflammatory effect [157]. Cytotoxicity of the same ZnO NPs to RKO human colon carcinoma cells was dependent on direct particle-cell contact and independent of the Zn^2+^ concentration in cell culture medium [158]. Exposure of the LoVo human colon carcinoma cell line to 50–70 nm ZnO NPs resulted in decreased viability, oxidative stress, depolarization of the inner mitochondrial membrane, apoptosis, and IL-8 release [159]. Another recent study also demonstrated time- and dose-dependent cytotoxicity of ZnO NPs on Caco-2 cells after 24 h exposure [160]. Here the authors also revealed that ZnO NPs exert different size-dependent cytotoxic effects with the highest toxicity to Caco-2 cells at 26 nm. These ZnO NPs at 26 nm could also reduce the G1 phase, increase the S phase and the G2 phase cells to repair damaged genes, while no differences were obtained between 62 nm and 90 nm ZnO NPs [160].

*In vivo* studies on mice have shown that the Zn concentration in the liver, spleen and kidney was higher after administration of zinc in nano form compared to similar amounts of ZnO microparticles. Oral NP administration resulted in transient histopathology of the liver that was not seen after administration of micro-sized ZnO particles [54]. The oral toxicity of ZnO NPs in mice revealed that NPs cause lung, liver and kidney damages [161]. Recorded significant increases in alanine and aspartate aminotransferases activity in all mice exposed to ZnO NPs suggest that these NPs can cause hepatic injury. These results were confirmed in another study [162]. Oral administration of 100 nm ZnO NPs (2.5 g/kg of body weight) resulted in their accumulation in the liver, spleen, lung, and kidney. In contrast to intraperitoneal administration, ZnO NPs did not accumulate in the heart [161]. More information on the existing literature on mammalian toxicity of ZnO NPs, both *in vitro* and *in vivo*, is nicely summarized in a recent review [163].

### 4.4. NanoSilica

Mesoporous SiO_2_ NPs have been extensively explored as effective drug delivery systems for a wide variety of therapeutic agents to fight against various kinds of diseases, e.g., tissue engineering, diabetes, inflammation and cancer [164,165,166,167]. Food-grade silica has traditionally been synthetic amorphous SiO_2 _produced in a variety of forms (pyrogenic, gel, sol, precipitate). Amorphous SiO_2_ has been used for many years in food applications, such as for clearing beers and wines, as anticaking agent to maintain flow properties in powder products and to thicken pastes [110], and as a carrier for fragrances or flavors in food and nanofood products [168,169]. The conventional form of SiO_2 _is known as the food additive E551. Dekkers *et al*. [111] estimated that the food products including E551 contain nanosilica ranged from <0.1–6.9 mg/g product based on the total silica concentration, where the particles sizes were ranging from 30–200 nm. The authors estimated that intake of SiO_2 _NPs based on consumption of food products analyzed for their nanosilica or silica concentration is about 124 mg/day. 

The abovementioned data, as well as growing biomedical and pharmacological applications of SiO_2_ NPs make an issue of current importance to investigate the influence of these NPs on the biological systems and on the GIT in particular. The *in vitro* cytotoxicity effects showed that SiO_2_ NPs (25 and 100 nm) induced a rather limited cytotoxic and genotoxic effects on HT-29 cells after 24 h exposure, with more expressed toxicity at lower concentrations [170]. From this study it could be concluded that SiO_2_ at 100 nm is more cytotoxic and genotoxic than SiO_2 _at 25 nm, but with an inverse relationship between effects and dose. The uptake following ingestion of SiO_2 _NPs cannot be excluded also given the reported oral toxicity data [110,168,171]. Particularly, it was shown that 10 weeks fed mice with amorphous SiO_2_ (30 nm, 140 g/kg mice) showed higher value of alanine aminotransferase activity than normal and micron-sized silica dieted groups [172]. Recently, it was concluded that single SiO_2_ NPs may be more easily absorbed from the human intestine [173], but that no conclusion on the oral bioavailability of synthetic amorphous silica or nano-sized silica can be drawn so far.

## 5. Health Implications of Nanofood, Consumer Safety Issues and Regulatory Aspects

Much of the debate about the safe use of nanotechnology in the food sector has focused on the uncertainties and the lack of toxicological data. At present, there is no tenable evidence and no general conclusion can be made on whether the food or food contact materials derived from nanotechnology are either safer or more dangerous than their conventional counterparts. Most scientific committees that have reviewed the applications of nanotechnology concluded that while consumers are likely to benefit from this technology, new data and new measurement approaches may be needed to ensure that the safety of products using nanotechnology are properly assessed [22,76,174,175]. The approaches for safety evaluation of ENMs vary from country to country but presumably follow similar pathways to those used for other materials proposed for use in food and food contact materials [176].

The information on the ENMs influence on the GIT is scarce. Recently, it was suggested that there could be an association between high levels of dietary NPs uptake and Crohn’s disease. Experimental results indicate that the accumulation of insoluble NPs in humans may be responsible for the compromised GI functioning, as described in the case of Crohn’s disease and ulcerative colitis [39]. Thus, an issue to be considered in relation to ENMs ingestion is the possible increase in their intestinal absorption in the case of systemic exposures, such as in Inflammatory Bowel Disease (IBD)—a chronic disorders characterized by recurrent and serious inflammation of the GIT [177]. In addition, the exposure to some NPs is associated with the occurrence of autoimmune diseases, such as systemic lupus erythematosus, scleroderma, and rheumatoid arthritis [6]. Moreover, nano- and microsized particles have been found to induce granulomas in different organs and tissues: inorganic particles heterogeneous in nature but homogeneous in size have been identified in liver and kidney biopsies from patients with granulomatosis of unknown origin (10–20 μm and 6–8 μm in the liver and kidney, respectively) [178]. Micro- and nanoparticles of non-biodegradable inorganic exogenous pollutants have been found in colon tissues affected by cancer and Crohn’s disease [179]. Concomitantly, both TiO_2_ (anatase) and aluminosilicate (as kaolinite) are commonly seen in the human intestinal lymphoid aggregates [180,181].

The exposure to ENMs may potentially lead to an immunomodulatory effect. Recent data indicate that systemic exposure to a single administration of ZnO NPs could enhance subsequent antigen-specific immune reactions, including the serum production of antigen-specific antibodies, and the functionality of T cells [182]. The immune effects of nano-TiO_2_ exposure were shown to be route-of-exposure dependent. After 28 days of oral gavage of mice with TiO_2 _NPs, irritancy and/or potential hypersensitivity responses were shown [183]. Exogenous NPs were found in macrophages accumulated in lymphoid tissue of the human gut, the lymphoid aggregates [39]. Microscopy studies showed that macrophages located in lymphoid tissue uptake NPs, such as spherical anatase (TiO_2_) with size of 100–200 nm from food additives, aluminosilicates in the 100–400 nm range, typical of natural clay, and environmental silicates of 100–700 nm with various morphologies [180]. All these data indicate that ingested ENMs have a potential to cause inflammatory response, lead to impaired mucosal immunity and thus to inflammation-associated diseases, e.g., Crohn’s disease and ulcerative colitis [39] or to induce allergic sensitization and result in allergic diseases, such as food allergy.

Recently, the results of studies conducted to assess the potential toxic effects of ENMs have suggested a number of adverse effects on human health such as alterations in the development and growth process, leading to the disruption of the endocrine system. The adverse effects of ENMs on the endocrine system are still unclear and unexplored and the potency of ENMs as endocrine disruptors should be taken into account. A recent review [184] summarizes and discusses recent reports derived from cell lines or animal models concerning the effects of ENMs on, and their application in the endocrine system of mammalian and other species. It presents an update on current studies of the effects of some typical NMs, e.g., metal- and carbon-based NMs, and dendrimers on endocrine functions, in which some effects are adverse or unwanted and others are favorable or intended. Studies of the reproductive function suggest that exposure to some ENMs may disrupt endocrine functions such as regulation of serum sex hormone levels. In contrast, other NMs may prevent endocrine dysfunction via various mechanisms, including antioxidant effects. Some recent studies presented evidence that metal-based ENMs, e.g., Ag, Au, Mn or Ti NPs, widely used in the consumer market, may exert endocrine-associated toxicity [183].

Another review [185] suggests that some of the ENMs may pose risks to male and female reproductive health by altering normal testis and ovarian structure, spermatogenesis and sperm quality, oogenesis, follicle maturation and sex hormone levels. Authors conclude that in the male reproductive system NPs are able to affect cell viability in gonadal tissues, testicular morphology and the process of spermatogenesis, whereas in the female reproductive system, NPs exert cytotoxic and/or genotoxic effects on ovarian structural cells and damage oogenesis and follicle maturation. Furthermore, NPs were able to cause significant alterations in normal sex hormone levels in both systems [185]. However, some studies failed to confirm these adverse effects, since no abnormalities in male and female reproductive function were found after NP exposure. These conflicting results are probably due to the different intrinsic properties of NPs used in the *in vitro* and *in vivo* studies.

From the available information so far it could be concluded that further investigations are required to obtain a thorough understanding of any potential risk to the GIT, as well as on systemic toxicity and pathological endocrine disruption in particular from products containing ENMs. Systematic biosafety assessment is necessary to evaluate the potential endocrine-disrupting risks of ENMs, which in turn is associated with adverse health outcomes including reproductive failure, metabolic syndrome, and some types of cancer.

Research on ingestion of ENMs as a direct exposure route is just beginning. The Food and Agriculture Organization and the World Health Organization [83] summarized information on the potential food safety implications of ENMs. According to this report, the assessment of exposures to ENMs poses challenges because of the need to characterize and quantify the material once it is released and to assess its stability and potential biotransformation during food processing or in food [83]. The crucial issues concern the propensity of ENMs to survive in the GIT (e.g., the acidic gastric environment), the ability and extent to be absorbed and assimilated in the organism. 

Another critical research question concerns the nature and implications of biomolecular modifications of ENMs in biological fluids and matrices. In particular, recent evidence indicates the formation of a “hard” corona with stable proteins and an outer, “weaker” corona that has quickly exchanging proteins depending on the environment [102,186]. Possible sources of surface coating/contamination of NPs are diverse. One example surface contaminant is ubiquitous bacterial endotoxins lipopolysaccharides (LPS)-one of the most common surface-adsorbed contaminants of serious concern for all biomaterials [187]. In pharmaceutical industries, it is possible to find endotoxins during production processes or in the final product [188]. It was shown that TiO_2_, ZnO and SiO_2_, upon their absorption and passage across the GIT adsorb calcium ions and LPS and the resulting NPs-calcium-LPS conjugates activate both peripheral blood mononuclear cells and intestinal phagocytes, which are usually resistant to stimulation [150]. Hence, endotoxin contamination is possible on a variety of NPs, producing an inflammatory response that is magnified by the NPs presence to levels that would warrant concern over endotoxin’s potent inflammatory reactivity *in vivo* and with many cell lines [189].

Another problem arising from the use of metals in food contact surfaces depends on the quantity of ions able to migrate into the food matrix. An intentional migration of the active element in the food matrix falls under Framework Regulation for active packaging materials [190]. When ENMs come in contact with food, indirect contamination can also be expected, if those NMs migrate. Guidance on the risk assessment of ENMs has been provided by the European Food Safety Authority (EFSA) on the potential risks arising from nanotechnologies [191]. In cases, where ENMs are not persistent, may migrate, be transformed before or during ingestion, *in vitro* and *in vivo* toxicity studies are recommended. The importance of the legislation concerning the use of metal-based ENMs is supported by recent observations. For instance, the quantification of the migration of Ag NPs and Ag^+^ in consumer products revealed that both Ag^+^ and NPs migrate at levels that approximate the expected toxicity in some goods [192]. EU safety regulations indicate that Ag zeolites in food contact applications should not be used to extend shelf-life, and the presence of Ag^+^ in food matrices is limited to 50 µg Ag^+^/kg food, which is not biocide in food [193]. The US FDA approved the use of Ag as an antimicrobial in bottled water, with a concentration up to 17 µg Ag^+^/kg [194]. The use of TiO_2_ as a colour additive approved by code E171 under is less restrictive and could be used in “*quantum satis*” [195].

Steps for the safety assessment for ENMs in foods are basically the same as those for conventional foods and follow the general risk assessment model [196,197]. As ENMs may be considered as “novel” forms of their bulk counterpart, it is insightful to consider the assessment processes developed for novel foods, as defined in Europe by Regulation (EC) No. 258/97 [198]. Currently in the process of the safety evaluation of novel foods, a comparative approach is used that makes use of existing data on a relevant non-novel comparator [199,200]. 

In the presence of a food matrix, in addition to the characterization and safety assessment of the ENMs as manufactured, it is important to characterize and achieve understanding of the form of the ENM, whether it is free or agglomerated, soluble or insoluble, coated or pristine, formulated or unformulated, *etc*. The latter is important, as in particular, the physicochemical characteristics of other ingredients/components within the food may influence the degree to which the ENM is digested or translocated in the GIT. Additionally, ENMs may be elaborated into a range of other structures that may either remain intact or act as delivery systems for the ENMs. Thus it could be concluded that the physicochemical characterization of the ENM as manufactured, in food matrices as consumed, and after exposure to biological matrices such as GIT fluid, mucus, plasma, lymph, therefore plays a pivotal role in guiding the safety assessment program. An adequate characterization of the ENMs under evaluation should be conducted at an early stage.

## 6. Discussion and Conclusions

Nanotechnology is a fast-growing field of activity that will allow development of materials with brand-new properties. In fact, as more and more consumer products containing ENMs become available on the market, the exposure of the general population will inevitably increase heightening the concern about the potential human toxicity and environmental impact of these particles. However, since there is still a lack of knowledge about the possible risks to human health and environmental safety posed by the expanding development and use of ENMs, there seems to be an urgent need to gather information on this subject. In this context, we have summarized the data currently available in the literature that report the adverse health effects of ENMs (mainly the metal (oxide) NPs) upon *in vitro* and *in vivo* exposure. Our aim was to understand the risk of food-associated ENMs that are ingested directly or indirectly via oral route, assess the underlying mechanisms that can affect the GIT function and identify research areas where further study needs to be carried out to reach a deeper understanding of the role of ENMs in food industry and potential consumer safety issues. 

The available data indicate that some insoluble ENMs can pass through the different protective barriers and the GIT barrier in particular, be distributed in the body, and accumulate in several organs. Toxic effects have already been documented at the pulmonary, cardiac, reproductive, renal, cutaneous, and cellular levels, while ENMs can be distributed throughout the body, including the interior of cells. Significant accumulations have been shown in the lungs, brain, liver, spleen, and bones. There are still big gaps in the understanding to what extent one or another type of ENM passes through the intestinal lining intact or is dissolved in the highly acidic environment of the stomach, and to what extent these ENMs can pass through natural biological barriers such as the gut epithelium, blood-brain barrier, the placenta or into breast milk. 

The hazard identification studies so far were conducted only on physiologically normal conditions, however, some diseased model conditions should be taken into account. For instance, it was revealed that diseases, such as diabetes, may lead to an increased absorption of particles in the GIT [201]. Thus more vulnerable members of the population, *i*.*e*., those with pre-existing digestive disorders, may potentially be more affected by the presence of ENMs, although, in contrast, ENMs may offer many potential routes to therapies for the same diseases. In addition, the presence of inflammation may enhance the translocation of ENMs into circulation [202,203].

Computational models should play a complementary role in allowing rapid prediction of potential toxicities of new and modified ENMs. These *in silico* methods, aiming at predicting the toxicity of ENMs, such as Quantitative Structure Activity Relationships (QSAR) are being investigated and could eventually provide a useful screening tool [204,205,206,207]. QSARs are theoretical models that relate the structure or physicochemical properties of substances to their biological activities. QSARs are being applied in many disciplines, for example in risk assessment, and toxicity prediction [208], as well as in drug discovery and lead optimization [209], but they have yet few applications for NPs (nano-QSAR) [210]. Recently nano-QSAR was applied to predict the cytotoxicity of metal oxide NPs [211], as well as predictive models of cellular uptake and apoptosis induced by NPs for several cell types was developed by Epa *et al*. [212]. The reliable QSAR models thus may provide a tool to predict the toxicity of ENMs, understand the link between the ENMs physicochemistry and the toxicity endpoints, as well as design safe nanomaterials for future biomedical and food applications.

Since the biological and toxic effects of ENMs are highly dependent on their physicochemical properties (size, shape, charge, coating, solubility *etc*.) as well as on dosage, route of administration, duration of exposure, *etc*. it becomes evident that a clear and precise evaluation of the biological effects of ENMs should also include a homogeneous exposure classification to ascertain exactly how the physicochemical properties of ENMs correlate with their adverse health effects. Useful parameters for a systematic categorization should include e.g., chemical composition, morphology, number concentration, surface area, mass concentration, weighted size distribution, state of agglomeration and surface reactivity (ability to produce radicals, zeta potential, *etc*.), as well as the surface (bio)functionalization when in contact with the surrounding matrix or biological media. This is fundamental for the assessment of the toxicity profiles of ENMs. Unfortunately much of this characterization is largely lacking in current studies due to the complicate nature of the characterization process (especially in biological fluids and food matrix). Better understanding of the processes underlying the interaction of ENMs with biomolecules will serve as a basis for biological and medical applications of ENMs.

Conducted toxicity studies aimed at identifying the potential risks of ENMs using various *in vitro* or *in vivo* assays frequently applied excessive and unrealistically high mass doses of ENMs and it is debatable whether the findings observed in these studies can be extrapolated to refer to humans in real life. It is therefore arises a need to study more realistic lower exposure scenarios both acute and chronic to have better relevance to the real-world conditions. Given the poor availability data on exposure levels of general population to food-associated ENMs, it is not easy to determine a realistic exposure dose to be used in the toxicological studies. In addition, the impact of traditional heat treatment against complement or other treatments (e.g., sonification) must all be addressed as important controls, if the results are to be meaningful for extrapolation to the *in vivo*, real consumer situation [213]. However, it seems clear that further studies need to focus on the potentially adverse effects of low-level ENM exposure with more emphasis on the matrix, where the ENMs actually are. Moreover, to immediate effects, time-series studies have shown cumulative effects over weeks, associated with elevated particle concentrations [214]. Hence, further studies are necessary to assess the health effects of chronic exposure to ENMs.
